# Women’s sexual empowerment and utilization of long-acting reversible contraceptives in Ghana: evidence from the 2014 demographic and health survey

**DOI:** 10.1186/s12905-023-02572-0

**Published:** 2023-08-09

**Authors:** Kenneth Setorwu Adde, Emmanuel Ayetey Appiah, Franklin N. Glozah, Philip T-N. Tabong

**Affiliations:** 1 Department of Population and Health, College of Humanities and Legal Studies, University of Cape Coast, Cape Coast, Ghana; 2 Department of Social and Behavioural Sciences, School of Public Health, University of Ghana, Accra, Ghana

**Keywords:** Contraceptives, Sexual empowerment, Sexually active, Pregnancy, Long-acting reversible contraceptives, Utilization, Women, Ghana

## Abstract

**Background:**

Long-Acting Reversible Contraceptives (LARC) contribute significantly to a decline in unintended pregnancies globally. However, not much is known about women’s sexual empowerment and their utilization of Long-Acting Reversible Contraceptives in Ghana. The main objective of this study was to examine the association between women’s sexual empowerment and LARC utilization in Ghana.

**Methods:**

We used data from 5116 sexually active women who participated in the 2014 Ghana Demographic and Health Survey. Women’s sexual empowerment was defined as women’s perception of their right to self-determination and equity in sexual relations, and their ability to express themselves in sexual decision-making. A sum of scores was created with four dichotomous items as sexual empowerment score (0 = low sexual empowerment; 1, 2, and 3 = medium sexual empowerment; and 4 = high sexual empowerment). Multivariable binary logistic regression analyses were performed to establish the association between women’s sexual empowerment and the use of LARC. Pearson Chi-square test was used in data analysis. The results are presented as adjusted odds ratios (aOR), with their respective confidence intervals (CIs) at a statistical significance of p < 0.05.

**Results:**

The prevalence of LARC utilization among sexually active women in Ghana was 6%. Majority of the women had medium sexual empowerment (91%). Although not statistically significant, the likelihood of utilizing LARC was lowest among women with high level of sexual empowerment (aOR = 0.62; CI = 0.27–1.43). On the other hand, Utilization of LARC increased with an increase in age. Women with parity four or more had higher odds of utilizing LARC as compared to women with zero birth (aOR = 9.31; CI = 3.55–24.39). Across religion, women who belong to the Traditional religion (aOR = 0.17; CI = 0.04–0.71) and Islam religion (aOR = 0.52; CI = 0.36–0.76) had lower odds of LARC utilisation as compared to Christian women. Women who make health decisions with someone else (aOR = 1.52; CI = 1.12–2.09) had higher odds of LARC utilisation as compared to women who make health decision alone.

**Conclusion:**

Age, health decision maker, parity and religion were found to have a significant relationship with LARC utilization. Specifically, uneducated women, unemployed women and women who practice traditional religion were less likely to utilise LARC. However, women’s sexual empowerment did not have a significant relationship with LARC. There is therefore the need for planning interventions for LARC utilization in line with educating women on the benefits and potential side effects of LARC. Also, there is a need for interventions targeted at increasing access to LARC among sexually active women.

**Supplementary Information:**

The online version contains supplementary material available at 10.1186/s12905-023-02572-0.

## Background

Women’s empowerment is a process of personal and social change through which they gain power by making meaningful choices, controlling and owning their lives. The pathways to women’s empowerment are numerous and include modern contraceptive use and medical procedures that interfere with sexual reproduction [1]. In addition to other contemporary contraceptive techniques, the World Health Organization (WHO) classifies implants and intrauterine contraceptive devices (IUCDs) as long-acting reversible contraceptives (LARCs). When compared to other contraceptive techniques, LARCs give a rapid return to childbearing capacity after removal and are extremely effective over an extended length of time. Additionally, they are accessible to all women of reproductive age [2]. There is also higher efficiency of LARCs over short-term contraceptive methods, and cost-effective after only one year of use. It is noted that the universal coverage for all women who require and are eligible for LARCs would generate a USD 120 billion annual return for each dollar spent. In Sweden and the United States of America, studies have demonstrated remarkable economic benefits of LARCs over short-term methods [3]. These benefits would have been more pronounced with the use of more effective LARCs, and more so if they had been provided in a single visit [4].

Women’s Empowerment is not something that can be done to and for them but they must be change agents of their empowerment. Among other things, the empowerment of women could include “the empowerment and autonomy of women and the improvement of their political, social, economic and health status” which is both a highly important end in itself and necessary for the achievement of sustainable human development and “advancing gender equality and equity, and the empowerment of women, and the elimination of all kinds of violence against women, and ensuring women’s ability to control their fertility are priority objectives of the international community” [5]. Furthermore, there is a need for a paradigm shift from population control to highlighting women’s reproductive and sexual rights and empowerment [6], which is grounded on the argument that across cultures, sexual relationships occur between individuals with unequal power [7, 8]. Consequently, women’s sexual empowerment is a significant factor that could promote female access to family planning. Maternal and child health care service utilization are significantly affected by women’s empowerment. The measurement of contraceptive use in several dimensions is important, considering the nature of empowerment processes as it relates to improvements in maternal health status [9]. The availability of different kinds of contraceptives and easy accessibility by women breeds confidence in their reproductive, maternal and child healthcare choices [10], thereby giving them some level of maternal independence in their health-seeking behaviour. This Maternal independence in healthcare-seeking behaviour in the view of Yaya, et al. [11], is connected to women’s empowerment and helps to achieve desired health outcomes. The more empowered women are, the more likely they are to use modern contraception, deliver in a health facility, and have a skilled attendant at birth [12]. Contraceptive use is important in preventing foetal, neonatal, and under-5 deaths; reducing maternal mortality and avoiding high-risk pregnancy including pregnancy among teenage girls and older women [11]. It is however worth noting that women’s empowerment is multidimensional, encompassing economic, sexual, political, and interpersonal, among others [12–15]. Whereas studies have focused mainly on the socio-economic aspects of women’s empowerment and contraceptive utilization [9, 11, 16] little is known about the influence of sexual empowerment of women on modern contraceptive utilization. For example, in Ghana, what has been examined focused on the association between women’s sexual empowerment and contraceptive use [17]. Also, although there is evidence that there is a higher efficacy for LARC as compared to Short-acting Contraception Methods – SAC [18, 19], evidence of the role of women’s sexual empowerment in the utilization of LARC is lacking. We, therefore, hypothesise that women’s sexual empowerment status predicts their utilization of LARC. Consequently, the current study, sought to examine the role of women’s sexual empowerment and LARC utilization in Ghana using the most recent Demographic and Health Survey (DHS) data. We also explored the effect of other covariates such as age, place of residence, wealth quintile, and employment status, [19–22] to account for other factors that are likely to influence utilization of LARC. Findings from this study could inform policymakers and all stakeholders to plan and design fit-for-purpose LARC utilization interventions and sexual empowerment strategies to accelerate the prospects of Ghana achieving SDG 3.1 and SDG 5.

## Materials and methods

### Data source

The study used data from the most recent Demographic and Health Survey conducted in Ghana in 2014. Specifically, data was pulled from the women’s files of the DHS data set. The 2014 Ghana Demographic and Health Survey aimed at providing current population-level estimates of basic demographic and health indicators. The survey collected data on health decision-making, fertility, awareness, and utilization of family planning methods, unintended pregnancy, contraceptive use, skilled birth attendance, and other essential maternal and child health indicators [23, 24].

The survey targeted women who were between 15 and 49 years old. The study used DHS data to provide in-depth evidence of the relationship between women’s sexual empowerment and the use of LARC in Ghana. DHS is a nationwide survey collected every five years across low- and middle-income countries. A stratified dual-stage sampling approach was used. The sampling procedure began with the identification of clusters (i.e. enumeration areas [EAs]) and then involved systematic household sampling within the chosen EAs. The existing DHS variable excluded women who were pregnant and those who had never had sex. For the purpose of this study, only women (15–49 years old) in sexual unions (marriage and cohabitation) with complete cases for all the relevant variables were considered in this study. The total sample for the study was 5,116.

### Study variables

#### Dependent variable

The dependent variable in this study was women’s self-report of currently using any “Long-Acting Reversible Contraceptive” which was derived from the ‘current contraceptive method’. The responses were coded 0 = “other methods”, and 1 = “Long-Acting Reversible Contraceptive”. The Long-Acting Reversible Contraceptive methods included intrauterine contraceptive device (IUD), and contraceptive implants (Norplant) [2]. Other methods on the other hand included female sterilization, contraceptive injection, contraceptive pills, condoms, emergency contraceptives, standard day method (SDM), vaginal methods (foam, jelly, suppository), lactational amenorrhea method (LAM), country-specific modern methods, and respondent-mentioned other modern contraceptive methods (e.g., cervical cap, contraceptive sponge). Other methods also included periodic abstinence (rhythm, calendar method), withdrawal (coitus interruptus), country-specific traditional methods of proven effectiveness, locally described methods and spiritual methods (e.g., herbs, amulets, gris-gris) of unproven effectiveness.

#### Explanatory variables

The main explanatory variable was women’s sexual empowerment. This was generated from a crude composite variable created to capture women’s perception of their right to self-determination and equity in sexual relations, and their ability to express themselves in sexual decision-making. The sum of scores on four dichotomous items was used as their sexual empowerment score (0 = low sexual empowerment; 1, 2, and 3 = medium sexual empowerment; and 4 = high sexual empowerment) [17, 25].

This was created from four items:

1. Can you say no to your husband/partner if you do not want to have sexual intercourse?
2. In your opinion, is a husband justified in hitting or beating his wife if she refuses to have sex with him?
3. Could you ask your husband/partner to use a condom if you want him to? and.
4. If a woman knows her husband has a disease that she can contract during sexual intercourse, is she justified in asking him to use a condom when they have sex?

In addition, some covariates were included based on theoretical relevance and conclusions drawn about their association with modern contraceptive utilization [19–22] These variables are age, place of residence (urban or rural), wealth quintile (poorest, poorer, middle, richer, richest), employment status (not working or working), educational level(no education, primary education, secondary or higher), marital status (Married or cohabiting), health decision-making capacity (respondent alone, respondent and someone, partner alone), and parity (zero birth, one birth, two births, three births, four births or more), religion (Christian, Islam, Traditionalist, no religion), frequency of reading newspaper (not at all or less than once a week), frequency of listening to radio (not at all or less than once a week) and frequency of watching television (not at all or less than once a week).

### Statistical analysis

We used STATA version 13 to analyse the data. We began by computing a descriptive analysis of LARC use in relation to women’s sexual empowerment and the covariates. We presented these as frequencies and percentages (Table 1). To assess the relationship between women’s sexual empowerment, covariates, and LARC use at a 5% margin of error, we used Pearson’s Chi-square tests (Table 2). Two models were computed. The first one focused on sexual empowerment and LARC, without any covariate (see Model I in Table 3). In the second model (Model 2 in Table 3), we adjusted for all the covariates described in the explanatory variables section. With crude odds ratios (cOR) with matching 95% confidence intervals, we provided the model’s findings. In the second model (Model II in Table 3), where adjusted odds ratios (aOR) were provided, we further investigated the influence of the variables to determine the overall impact of women’s sexual empowerment on the use of LARC. As reference categories for the independent variables, normative categories were used. In order to get findings that are representative at the national and domain levels, sample weighting was used for computing the frequencies and percentages. To account for the survey’s complicated sampling process, we employed STATA’s survey command (svy) in the regression models. We assessed multicollinearity among our co-variates with Variance Inflation Factor (VIF) and realized that no multicollinearity existed with a mean VIF = 5.04. The model was further validated with a firth logistic regression to ensure that the model that was not significant, from the analysis, was not as a result of the skewed nature of the data [26]. In addition, we performed a goodness of fit test and we recorded a P-value of 1.00 which indicates that the test is not significant and that our model fits well (see Additional File Table 1).

### Ethical approval

This study used publicly available data from DHS, however, the actual study (2014 GDHS) received ethical approval from the Ghana Health Service Ethics Review Committee Informed consent was obtained from all participants prior to the survey. The DHS Program adheres to ethical standards for protecting the privacy of respondents. The ICF International also ensures that the survey processes conform to the ethical requirements of the U.S. Department of Health and Human Services. No additional ethical approval was required, as the data is secondary and available to the general public. However, to have access to and use the raw data, we sought and obtained permission from MEASURE DHS. Details of the ethical standards are available at http://goo.gl/ny8T6X.

## Results

### Background characteristics of women and the utilization of LARC in Ghana

From Table 1, the highest proportion of women (91.1%) have a level of medium sexual empowerment. The majority of the women were aged 30–34 years and a larger proportion had secondary or higher education. About half (50%) of the women reside in Urban areas and 74.3% were married. A higher proportion of the women were working (87.3%), in the richest wealth status (23.4%), and making health decisions with someone (50.2%). A high proportion of the women have 4 + children (43.1%) and were Christians (77.5%). Regarding the media variables, a higher proportion (87%) do not read newspapers or magazines at all, listen to the radio less than once a week (85%) and watch television less than once a week (74%). The prevalence of LARC utilization was 6% (298).

**Table 1:** Background characteristics of respondents

Variable	Frequency (n = 5116)	Percentage (%)
**Sexual Empowerment**		
Low	129	2.52
Medium	4658	91.05
High	329	6.43
Total		
**Age**		
15–19	100	1.96
20–24	585	11.44
25–29	1014	19.82
30–34	1040	20.34
35–39	1013	19.80
40–44	777	15.18
45–49	587	11.46
Total		
**Level of education**		
No education	1389	27.15
Primary	936	18.30
Secondary or higher	2791	54.55
**Place of residence**		
Urban	2563	50.09
Rural	2553	49.91
**Marital status**		
Married	3803	74.34
Cohabiting	1313	25.66
**Occupation**		
Not working	650	12.70
Working	4466	87.30
**Wealth index**		
Poorest	942	18.42
Poorer	932	18.22
Middle	979	19.14
Richer	1068	20.87
Richest	1195	23.35
**Health decision maker**		
Respondent alone	1413	27.61
Respondent and someone	2567	50.18
Partner alone	1136	22.21
**Parity**		
Zero birth	327	6.39
One birth	749	14.65
Two births	922	18.01
Three births	912	17.82
Four births or more	2206	43.13
**Religion**		
Christian	3964	77.47
Islam	853	16.67
Traditionalist	133	2.59
No religion	167	3.27
**Frequency of reading newspaper or magazine**		
Not at all	4441	86.80
Less than once a week	675	13.20
**Frequency of listening to radio**		
Not at all	777	15.18
Less than once a week	4339	84.82
**Frequency of watching television**		
Not at all	1339	26.17
Less than once a week	3777	73.83

**Table 2:** Long-acting reversible contraceptive prevalence by socio-demographic characteristics

Socio-demographic characteristics	Type of Contraceptive		*X*^*2*^; *p-value*
	Other Methods n (%)	LARC n (%)	
**Sexual Empowerment**			
Low	127 (93.38)	9 (6.62)	X^2^ = 1.1043; p = 0.576
Medium	4325 (94.10)	271 (5.90)	
High	366 (95.31)	18 (4.69)	
**Total**	**4818**	**298**	
**Age**			
15–19	108 (94.74)	6 (5.26)	X^2^ = 7.62; p = 0.267
20–24	588 (94.23)	36 (5.77)	
25–29	957 (93.55)	66 (6.45)	
30–34	933 (92.84)	72 (7.16)	
35–39	933 (94.53)	54 (5.47)	
40–44	737 (95.10)	38 (4.90)	
45–49	562 (95.58)	26 (4.42)	
**Total**	**4818**	**298**	
**Level of education**			
No education	1657 (95.28)	82 (4.72)	X^2^ = 9.0002; p = 0.011
Primary	899 (92.49)	73 (7.51)	
Secondary or higher	2262 (94.05)	143 (5.95)	
Total	4818	298	
**Place of residence**			
Urban	2206 (95.09)	114 (4.91)	X^2^ = 6.423; p = 0.011
Rural	2612 (93.42)	184 (6.58)	
Total	4818	298	
**Marital status**			
Married	3728 (94.36)	223 (5.64)	X^2^ = 1.0330; p = 0.309
Cohabiting	1090 (93.56)	75 (6.44)	
Total	4818	298	
**Occupation**			
Not working	645 (95.70)	29 (4.30)	X^2^ = 3.279; p = 0.070
Working	4173 (93.94)	298 (6.06)	
Total	4818	298	
**Wealth status**			
Poorest	1331 (94.53)	77 (5.47)	X^2^ = 8.4877; p = 0.075
Poorer	928 (92.80)	72 (7.20)	
Middle	904 (93.39)	64 (6.61)	
Richer	844 (94.62)	48 (5.38)	
Richest	811 (95.64)	37 (4.36)	
Total	4818	298	
**Health decision maker**			
Respondent alone	1206 (95.56)	56 (4.44)	X^2^ = 15.7072; p = 0.000
Respondent and someone	2552 (92.97)	193 (7.03)	
Partner alone	1060 (95.58)	49 (4.42)	
Total	4818	298	
**Parity**			
Zero birth	282 (98.26)	5 (1.74)	X^2^ = 25.4123; p = 0.000
One birth	728 (96.81)	24 (3.19)	
Two births	845 (94.31)	51 (5.69)	
Three births	807 (93.84)	53 (6.16)	
Four births or more	2156 (92.89)	165 (7.11)	
Total	4818	298	
**Religion**			
Christian	3470 (93.20)	253 (6.80)	X^2^ = 24.7936; p = 0.000
Islam	1006 (93.20)	253 (6.80)	
Traditionalist	158 (98.55)	36 (3.45)	
No religion	184 (96.34)	7 (3.66)	
Total	4818	298	
**Frequency of reading newspaper or magazine**			
Not at all	4290 (94.14)	267 (5.86)	X^2^ = 0.0892; p = 0.765
Less than once a week	528 (94.45)	31 (5.55)	
Total	4818	298	
**Frequency of listening to radio**			
Not at all	807 (94.50)	47 (5.50)	X^2^ = 0.1930; p = 0.660
Less than once a week	4011 (94.11)	251 (5.89)	
Total	4818	298	
**Frequency of watching television**			
Not at all	1602 (94.18)	99 (5.82)	X^2^ = 0.0001; p = 0.992
Less than once a week	3216 (94.17)	199 (5.83)	
Total	4818	298	

### Association between women’s sexual empowerment and utilization of LARC

From Table 3, although both the unadjusted and adjusted models were not statistically significant, the results showed that the utilization of LARC decreases with an increase in women’s sexual empowerment (OR = 0.69; CI = 0.30–1.58) and (aOR = 0.62; CI = 0.27–1.43) respectively. This we further confirmed with a firth logistic regression given that the prevalence of LARC utilization was low (see Additional File Table 2). We also observed that the odds of utilising LARC reduce with an increase in age. Also, working women have higher odds of using LARC as compared to women who are not working (aOR = 1.46; CI = 0.97. – 2.21). In addition, women who make health decisions with someone had higher odds to use LARC as compared to their counterparts who make health decisions alone (aOR = 1.52; CI = 1.12–2.09). We further observed that utilization of LARC increases with an increase in childbirth while traditional women had the lowest odds of using LARC when compared with their Christian counterparts (aOR = 0.17; CI = 0.04–0.71). Concerning the media variables, women who read newspaper or magazine less than once a week and women who watch television less than once a week had a higher odds of using LARC (aOR = 1.17; CI = 0.76–1.78) and (aOR = 1.17; CI = 0.85–1.61) respectively as compared to their counterparts who do not read newspaper/magazine or watch television at all. Finally, women who listen to radio less than once a week had a lower odds of using LARC as compared to those who do not listen to radio at all (aOR = 0.97; CI = 0.69–1.36).

**Table 3:** Multivariable logistic regression results on the predictors of LARC utilization among women in Ghana

Variables	Model 1	Model 2
**Sexual Empowerment**		
Low	Ref	Ref
Medium	0.88 [0.44–1.77]	0.81 [0.40–1.63]
High	0.69 [0.30–1.58]	0.62 [0.27–1.43]
**Age**		
15–19		Ref
20–24		0.75 [0.30–1.88]
25–29		0.56 [0.27–1.42]
30–34		0.45 [0.17–1.15]
35–39		0.29** [0.11–0.76]
40–44		0.23** [0.09–0.64]
45–49		0.22** [0.08–0.60]
**Level of education**		
No education		Ref
Primary		1.36 [0.96–1.94]
Secondary or higher		1.25 [0.88–1.77]
**Place of residence**		
Urban		Ref
Rural		1.06 [0.76–1.48]
**Marital status**		
Married		Ref
Cohabiting		1.04 [0.77–1.40]
**Occupation**		
Not working		Ref
Working		1.46 [0.97–2.21]
**Wealth status**		
Poorest		Ref
Poorer		1.07 [0.74–1.53]
Middle		1.04 [0.68–1.58]
Richer		0.97 [0.58–1.61]
Richest		0.82 [0.45–1.49]
**Health decision maker**		
Respondent alone		Ref
Respondent and someone		1.52** [1.12–2.09]
Partner alone		1.11 [0.74–1.66]
**Parity**		
Zero birth		Ref
One birth		1.92 [0.72–5.11]
Two births		4.11** [1.61–10.57]
Three births		5.15** [1.97–13.44]
Four births or more		9.31*** [3.55–24.39]
**Religion**		
Christian		Ref
Islam		0.52** [0.36–0.76]
Traditionalist		0.17* [0.04–0.71]
No religion		0.49 [0.23–1.08]
**Frequency of reading newspaper or magazine**		
Not at all		Ref
Less than once a week		1.17 [0.76–1.78]
**Frequency of listening to radio**		
Not at all		Ref
Less than once a week		0.97 [0.69–1.36]
**Frequency of watching television**		
Not at all		Ref
Less than once a week		1.17 [0.85–1.61]

Computed from 2014 Ghana Demographic and Health Survey *Ref* reference category.

*p < 0.05; **p < 0.01; ***p < 0.001.

## Discussion

This study examined the influence of women’s sexual empowerment on the utilization of long-acting reversible contraceptives among sexually active women in Ghana. We also examined how socio-demographic characteristics interact with women’s empowerment to predict LARC utilization. There was a low prevalence of LARC utilization (6%) among sexually active women in Ghana which corroborates the findings of Adedini, Omisakin and Somefun [27] who also observed a low prevalence of 4% in Ghana. Our results showed that the likelihood of utilising LARC was lower among women with high sexual empowerment. It is however worth noting that this was not statistically significant. A possible explanation is that these women, although sexually empowered may fear side effects and therefore opt against the use of LARC. This affirms the findings of Rominski et al. [28] who observed that at least 70% of women who chose IUD or implant as a method of family planning expressed, they will discontinue if they experience any side effects. Rominski et al. [28] also observed that these women’s choice of family planning method usually matches the duration of effectiveness but not the potential side effects, with a higher proportion of the women reporting not being counselled on the side effects of their chosen method. There is therefore a need for family planning clinics to provide counselling on the side effects of contraceptive methods. A better understanding of the side effects will reduce the discontinuation of modern contraceptive methods [28, 29].

Also, the odds of using LARC reduce with an increase in age. This finding resonates with Adebowale et al. [30] and Ahinkorah et al. [31]. A possible explanation is that older women might be less sexually active as compared to younger women who do not intend to birth children or have unplanned pregnancies. It is therefore not surprising that we found women with primary education having a higher likelihood of using LARC as compared to their counterparts with no education. Another reason why older women were less likely to use LARC could be that older women are less sexually active because as women age, they are likely to lose their partners to death, divorce, or separation [32]. Similarly, we found that working women were more likely to utilise LARC as compared to unemployed women. A plausible explanation could be that this group of women do not want pregnancy to interfere with their work, so they would opt for LARC to enable them to focus on their work without having to worry about unintended pregnancy. The direct and indirect costs of accessing LARC could serve as a barrier to unemployed women utilising LARC [30, 33–35]. Surprisingly, we observed the lowest odds for utilising LARC among women in the richest wealth quintile which corroborates the findings of Nyarko [35] who also observed lower odds for modern contraceptive utilization among women from rich households. This may possibly be a result of the fact that poorer sexually active women may want to avoid unintended pregnancies until they can afford to have a baby. Whereas the richer sexually active women could afford to have a baby and do not require the use of LARC and hence could rely more on short-acting reversible contraceptives. The use of LARC has also been proven to be more cost-effective as compared to SAC [36, 37] which could further explain why poorer sexually active women appear to be more likely to utilise LARC.

In this current study, we also observed that women who make health decisions with someone else are associated with increased odds of using LARC. This corroborates findings from Burkina Faso [38] and Mozambique [39]. This provides notable evidence that spousal influence has a positive effect on the utilization of LARC which may be down to patriarchal system practices in Ghana, which highlights the fact that there is a need to empower women to have autonomy over their health and contraceptive decisions. We also observed a positive association between parity and the utilization of LARC. The utilization of LARC increases with an increase in childbirth which affirms findings from previous studies [23, 40]. This is however expected since multiparous women may want to reduce their chances of having unplanned pregnancies.

Finally, regarding religion, we found that traditional women had the least likelihood of using LARC and Christian women had the highest likelihood of using LARC. Previous studies by Unumeri et al. [41] and Nyarko [35] also observed that Christian women have higher odds of using modern contraceptives. This could be attributed to the fact that pronatalist norms and doctrines are the key determinants in reproductive health decision-making for these religions [42].

### Strength and limitations

This study employed a nationally representative dataset consisting of 5116 women between the ages of 15 and 49, we are confident in the findings of the study. This study’s conclusions are also supported by the use of a quite robust statistical technique - logistic regression. Despite these strengths, the study has several weaknesses. First, the cross-sectional design used in the study restricts the ability to draw conclusions about the causal relationship between respondents’ individual characteristics and LARC utilization. Second, we agree that the significant variables in the model may have mediated the relationship between women’s sexual empowerment and LARC use. This notwithstanding, we strongly think that the large differences in frequencies between the categorisation of LARC into Low, Medium, and High may have contributed to this result and that performing a mediation analysis would yield a similar result. Thus, we did not conduct a mediation analysis and hence could not confirm if the factors that were significant could have mediated the relationship between women’s sexual empowerment and LARC use. There is also a chance that social desirability and data memory bias would have affected the findings because most of the questions were self-reported. Additionally, because only women participated in this study, partners’ viewpoints are not included in the results. Also, the composite variable (women’s sexual empowerment) was created into three groups based on the sum of scores. It is possible that an arbitrary grouping may not reflect the underlying distribution of the data. It is also worth noting that the 2014 GHDS which is the most recent for Ghana is nearly a decade old, hence findings from this study should be considered in that regard. Finally, factors such as cultural norms and attitudes of healthcare providers may have the potential to affect the outcomes of the study but these were not explored in this study, as these are not included in the DHS dataset.

## Conclusion

Long-acting reversible contraceptive utilization among sexually empowered women is low in Ghana. Socio-demographic characteristics play an important role in the utilization of LARC among sexually active women in Ghana. Specifically, uneducated women, unemployed women, women who practice traditional religion and women who make health decisions alone are unlikely to utilise LARC. The findings also demonstrate that age, health decision maker, parity and religion have a significant association with LARC utilization. However, women’s sexual empowerment did not have a significant relationship with LARC. This we explained could be attributed to the fact that although women could be sexually empowered, they might lack knowledge of LARC thus resulting in the fear of side effects. This research highlights the need for pragmatic steps to be taken to empower women by increasing sex education through awareness creation and the importance of LARCs. This would increase the chances of Ghana achieving SDG 3, by increasing the prevalence of LARC among sexually active women by providing universal access to family planning services and information to uneducated women, cohabiting women and unemployed women. Finally, family planning programmes and policies should also target these groups of women.

### Electronic supplementary material

Below is the link to the electronic supplementary material.

**Additional File 1:** Table 1 and Table 2

## Data Availability

Data used for the study is freely available to the public via https://dhsprogram.com/data/available-datasets.cfm.

## Abbreviations

LARC: Long-Acting Reversible Contraceptive
aOR: Adjusted Odds Ratio
GDHS: Ghana Demographic and Health Survey
cOR: Crude Odds Ratio
DHS: Demographic and Health Survey
EAs: Enumeration Areas
IUD: Intrauterine Device
LAM: Lactational amenorrhea method
LMICs: Lower-and middle-income countries
LR: Likelihood Ratio
PSU: Primary Sampling Unit
SDM: Standard Day Method
SRHR: Sexual and Reproductive Health Right
SSA: Sub-Saharan Africa
VIF: Variance Inflation Factor
WHO: World Health Organisation
