# Supplementary material for: Women’s sexual empowerment and utilization of long-acting reversible contraceptives in Ghana: evidence from the 2014 demographic and health survey
Source: BMC Womens Health. 2023 Aug 9;23:421. doi: 10.1186/s12905-023-02572-0 (PMC10413617; doi:10.1186/s12905-023-02572-0)
Supplement: Supplementary file 1 — Additional File 1: Table 1 and Table 2 [file 12905_2023_2572_MOESM1_ESM.docx]

**Supplementary File 1**

1. **Table 1: Goodness of fit test to test logistic model**

|  |  |
| --- | --- |
| Number of observations | 5116 |
| Number of covariate patterns | 3576 |
| Pearson chi2 (3546) | 3175.06 |
| Prob > chi2 | 1.00 |

1. **Table 2: Validation of logistic model with firth logistics regression**

| **Variables** | **Model 1** | | | **Model 2** | | |
| --- | --- | --- | --- | --- | --- | --- |
| **Women’s sexual empowerment** | **Coef** | **P-Value** | **[95% CI]** | **Coef** | **P-Value** | **[95% CI]** |
| Low | Ref |  |  | Ref |  |  |
| Medium | -0.17 | 0.616 | [-0.84 - 0.50] | -0.26 | 0.457 | [-0.95 – 0.43] |
| High | -0.39 | 0.345 | [-1.20 – 0.42] | -0.50 | 0.233 | [-1.32 – 0.32] |
